# Neuromarketing and consumer neuroscience: contributions to neurology

**DOI:** 10.1186/1471-2377-13-13

**Published:** 2013-02-06

**Authors:** Andrija Javor, Monika Koller, Nick Lee, Laura Chamberlain, Gerhard Ransmayr

**Affiliations:** 1 Department of Neurology and Psychiatry, Linz General Hospital, Krankenhausstrasse 9, 4021, Linz, Austria; 2 Department of Marketing Institute for Marketing Management, Vienna University of Economics and Business, Augasse 2-6, Vienna, 1090, Austria; 3 Aston Business School, Aston University, Aston Triangle, Birmingham B4 7ET, United Kingdom

**Keywords:** Neuromarketing, Neurology, Consumer neuroscience, Trust, Reward, Ethics, Pathological gambling, Compulsive buying

## Abstract

**Background:**

‘Neuromarketing’ is a term that has often been used in the media in recent years. These public discussions have generally centered around potential ethical aspects and the public fear of negative consequences for society in general, and consumers in particular. However, positive contributions to the scientific discourse from developing a biological model that tries to explain context-situated human behavior such as consumption have often been neglected. We argue for a differentiated terminology, naming commercial applications of neuroscientific methods ‘neuromarketing’ and scientific ones ‘consumer neuroscience’. While marketing scholars have eagerly integrated neuroscientific evidence into their theoretical framework, neurology has only recently started to draw its attention to the results of consumer neuroscience.

**Discussion:**

In this paper we address key research topics of consumer neuroscience that we think are of interest for neurologists; namely the reward system, trust and ethical issues. We argue that there are overlapping research topics in neurology and consumer neuroscience where both sides can profit from collaboration. Further, neurologists joining the public discussion of ethical issues surrounding neuromarketing and consumer neuroscience could contribute standards and experience gained in clinical research.

**Summary:**

We identify the following areas where consumer neuroscience could contribute to the field of neurology:

First, studies using game paradigms could help to gain further insights into the underlying pathophysiology of pathological gambling in Parkinson’s disease, frontotemporal dementia, epilepsy, and Huntington’s disease.

Second, we identify compulsive buying as a common interest in neurology and consumer neuroscience. Paradigms commonly used in consumer neuroscience could be applied to patients suffering from Parkinson’s disease and frontotemporal dementia to advance knowledge of this important behavioral symptom.

Third, trust research in the medical context lacks empirical behavioral and neuroscientific evidence. Neurologists entering this field of research could profit from the extensive knowledge of the biological foundation of trust that scientists in economically-orientated neurosciences have gained.

Fourth, neurologists could contribute significantly to the ethical debate about invasive methods in neuromarketing and consumer neuroscience. Further, neurologists should investigate biological and behavioral reactions of neurological patients to marketing and advertising measures, as they could show special consumer vulnerability and be subject to target marketing.

## Background

Scientific development in recent years is characterized by an expansion in the application of different and multidisciplinary research modalities in order to answer the various questions of a given scientific field. Of particular interest is the explosion in the use of neuroscientific methods, ostensibly to better understand human behavior in various contexts. This has led to the creation of the term ‘neuroculture’ [1], to refer to new scientific branches combining neuroscience with other scientific branches, arts or humanities, examples of this phenomenon include neurophilosophy (e.g. [2]) or neurotheology (e.g. [3]). Similarly, but in an even more pejorative sense, Tallis [4] coined the term ‘neuromania’ to refer to a headlong rush by seemingly all fields of study to embrace neuroimaging, and explain all human phenomena in terms of brain activity. Yet while Tallis [4], and others critical of the embracing of neuroimaging by various fields of study present powerful caveats against the unquestioning acceptance that human life in all its complexity can be reduced to brain activity, it is undeniable that – when applied properly – neuroimaging has much to offer as an addition to existing scientific tools, techniques, and frameworks [5].

Of particular interest is brain imaging research in the economic and business disciplines. Indeed, economists were amongst the first social scientists to recognize the potential of neuroimaging, with the development of neuroeconomics [6-12]. Soon after the first neuroeconomic papers had been published, marketing scholars discovered the potential of neuroscientific methods as a new research approach beside the classical qualitative and quantitative methodological spectrum in the social sciences. Early on in the field’s development, the term ‘neuromarketing’ research was suggested in order to categorize studies in the interdisciplinary field between economics, psychology, biology and medicine [13]. However, in the ensuing decade, multiple definitions of neuromarketing appeared to develop, even as neuroscientific and radiological advances expanded the array of tools available to researchers in this fledgling field [14-16].

Generally speaking, neuroscientific methods are used to study consumer behavior and the decision-making processes in purchasing acts [17], to better understand psychological phenomena and emotions in purchase decisions, as well as provide a more comprehensive assessment of the efficacy of marketing phenomena like advertising, consumer competitions, and product placement, by analyzing the underlying neurobiology [18]. Such studies are per se purely academic, although they clearly try to develop and derive recommendations for practical marketing. Independently, there are many businesses offering neuroscientific methods under the umbrella term ‘neuromarketing’. Often, the services offered by such firms, and their explanatory power, appear exaggerated in advertising and one only has to have a cursory understanding of neurophysiology to see that many of the outlandish claims made in the popular press about how neuroimaging can identify complex phenomena such as ‘love’, or behaviors such as purchasing, to be far overblown [19]. A recent study identified over 150 such companies [20]. An analysis of 16 companies identified through an internet search revealed that 5 of them offered fMRI, 9 EEG and 12 Galvanic-Skin response and other tests of the autonomic nervous system as methods [21].

Of course, the application of neurophysiological methods as adjuvant instruments to behavioral data in marketing research is not entirely new, but has attained media presence in last decade by the use of easily-discussed imaging methods such as functional magnetic resonance imaging (fMRI), and the special influence that brain images have on non-neuroscientists [22]. In 2008, Hubert and Kenning reported more than 800,000 Google hits for the term ‘neuromarketing’ [23], and in 2012, the same search yields over 1.4 million hits, underlining the continuing interest in this topic.

This development has further led to a discussion about ethical aspects of neuromarketing not only in scientific communities, but also in the general media [24,25]. Suddenly, journalists and by extension consumers appear to fear that market researchers might be able to analyze their private thoughts and emotions during purchase, and even be able to influence them to buy (e.g. [26]). This fear is not new though, and appears to be broadly similar to earlier fears over subliminal advertising (for an overview see [27]), even though this idea was later debunked (e.g. [28]). Of course, traditional market research has always been interested in analyzing and predicting purchase behaviors, but the advent of high-profile neuroimaging studies seems to have driven an explosion in public attention. Given the lack of knowledge on how experimental studies including marketing research are in fact performed, the general public are easily frightened. These ethical concerns have been further inflamed by the foundation of more and more enterprises (especially in the United States) offering neuromarketing as a service.

Unfortunately, debates over neuromarketing tend to lack a differentiation between scientific and commercial for-profit applications [29]. Especially in the public ethical discussion, it is important to distinguish academic studies that use neuroscientific methods from those purely for the purposes of commercial marketing. It is not taken into consideration, that scientific studies often focus on the consumer’s point of view, while commercial ones try to apply findings in order to sell a product. In particular, human beings do not act in a vacuum, and human behaviors are almost always context-laden. Much human behavior occurs within a consumption context, and it has been argued that incorporating such a context to neuroscientific work can also be of significant benefit [5,19,29]. Academic studies in neuromarketing have a highly interdisciplinary character. Knowledge from marketing management is tied together with psychological knowledge and different medical fields (above all neurology, psychiatry and radiology). As the general media mostly address commercial marketing when reporting about neuromarketing, it is essential to decouple these two entities and to controvert the mechanistic public opinion about the brain-behavior relationship by terming the scientific branch ‘consumer neuroscience’ [14,19,20]. A similar debate about adding the ‘neuro-’prefix to other behavioral sciences and the consequences of this ‘neuromania’ has been led lately by several authors in leadership research [14,19,30].

Furthermore, among scientists and journalists, there is an ambiguous view of neuromarketing. Beside a group of advocates, who represent the opinion that neuromarketing would lead to product improvement and therefore is beneficial for consumers [24,25], there are numerous critics [31-33]. For example, an editorial in ‘Nature Neuroscience’ stated that: ‘Neuromarketing is little more than a new fad exploited by scientists and marketing consultants to blind corporate clients with science.’ [34,35]. Hence, we strongly suggest a discussion of both what neuromarketing can and cannot do, and also what it should and should not do, involving experts from both business and neuroscientific research as well as ethicists and philosophers. Willingham and Dunn [36] elaborated ways of integrating brain imaging data into social psychology theory. A similar synthesis of brain imaging data and marketing theory still has to be developed. The results of such a discussion could then be presented to the public and would lead to an informed public view of neuromarketing.

Research in the field of what was termed above ‘consumer neuroscience’ has on the other hand generally been positively accepted within the academic community. However, while marketing scholars have eagerly integrated neuroscientific evidence into their theoretical frameworks, medicine is reluctant to adopt the results of consumer neuroscience. First attempts to transfer knowledge between neuroeconomics and psychiatry have been recently published [37], but it is clear that a joint discussion of how knowledge gained in ‘consumer neuroscience’ can contribute to a broader field of science, including especially biology, neuroscience, psychiatry and neurology, is still scarce. Despite this, findings from consumer neuroscience studies are significantly contributing to all behavioral sciences, especially by focusing on the interaction of cognitions and emotions in human behavior [38-40].

## Discussion

As such, the results of consumer neuroscience research can be fruitful for both scientific and clinical neurology for a number of reasons. Behavior has always been a major topic in neurological and psychiatric research and has led to the subspecialty of behavioral neurology, which manages the diagnosis of, and therapy for, behavioral symptoms of neurological disorders, e.g. dementia, depression, psychosis, anxiety, obsessive-compulsive disorder, attention deficit/hyperactivity disorder, autism, and agitation through neuropsychological and neurophysiological methods (including neuroimaging) [41]. Further, human behavior and the activities of daily living (ADL) are included in several diagnostic classifications and rating scales (e.g. ICD-10, DSM IV, Schwab and England activities of daily living scale). Consumer behavior research is considered a behavioral science and studies humans in these daily activities and in real-world settings. Neuroeconomics and consumer neuroscience investigate neural correlates of human behavior related to job performance, social and consumption behavior. Behavioral neurology may obtain a more comprehensive understanding of human behavior by incorporating insights from interdisciplinary approaches like consumer neuroscience, that analyze behavior relevant to the real world (see also [42]).

Consumer neuroscience has so far studied a large number of neurobiologically-oriented topics, and as such a complete review would be out of our present scope. Rather, we chose to concentrate on three topics that appear to be of most relevance to neurologists. We focus on (1) the reward system and its relations to brand preference and decision-making in purchasing, as it plays an important role in several neurological diseases and their behavioral symptoms. Further, we explore (2) the neurobiology of trust, as it is the basis of every patient-physician relationship and therefore of special interest to any physician. Finally, we include a discussion of (3) the ethical aspects of neuromarketing, since they dominate public debate in this field.

The three key topics will be briefly summarized and discussed in terms of their relevance to neurology in subsequent sections of this paper. In doing so, we try to make clear that the ecologically-valid research settings available in marketing research contexts can add significantly to neurology. As such, on the one hand, the findings of consumer neuroscience that we present in this paper might initiate further neurological and clinical research in an interdisciplinary setting. On the other hand, neurologists might be increasingly interested in joining the public discussion about neuromarketing and its ethical concerns. Both outcomes would be of significant benefit to both scientific research and general social progress. The paper aims at providing the basis for an enhanced two-way discussion instead of the one-way path currently active.

### Consumer neuroscience and the reward system

Two major brain systems are considered to be fundamental to almost all human behavior; the reward approach (pleasure-seeking) and the loss/pain avoidance systems [43,44]. The neurobiology of the reward system is based on the meso-limbic pathway, which extends from the ventral tegmental area (VTA), through the nucleus accumbens (NACC) and the limbic system, to the orbitofrontal cortex (OFC), while anticipation of loss, pain or punishment activates the insula [45-48], for a review see [49,50]. Differential roles for these brain areas have been recently detected and are summed up as the ‘Schultz Theory’ [51]. The nucleus accumbens seems to play a role as an integration site, receiving impulses from the OFC, which represents reward expectations, the amygdala (responsible for reward conditioning), and dopamine neurons, all of which play a role in reward prediction. The VTA and substantia nigra show a high density of dopaminergic neurons. Brain stimulation studies have shown that activation of these dopaminergic systems leads to feelings of ‚well being’ [52]. Outputs of the striatum to the VTA code for mismatch between predictors and reward.

Behavior is closely related to the reward system. In animals, basic rewards like food, drink and sexuality are predominant goals to be achieved through behavior. However, in humans more abstract forms like financial and social rewards (success, social status, culture etc.), or drugs that interfere with the neurophysiology of the reward system, are also main targets. In addition, certain physical objects, like cars [53] or money [54] can be rewarding. There is a considerable inter-individual variance in the sensitivity to reward stimuli [55]. In classical marketing as well as in consumer neuroscience, major research topics include the purchasing act and how this behavior is influenced, for example by the preference for a certain brand, although *how* brands effect consumer decisions is still a matter of debate [56-58].

The neurobiological basis of brand preference has been a research topic from the beginning of consumer neuroscience, and remains so today. The dorsolateral prefrontal cortex (DLPFC) is thought to be prominently active in the representation and integration of goals and reward information [59] and might initiate, through connections to the mesolimbic system, reward-motivated behavior [60]. The function of the ventromedial prefrontal cortext (VMPFC) is however still debated in neuroscience literature. Most authors suggest an important role in decision-making, especially in choice tasks [61].

In consumer neuroscience the VMPFC is studied in the context of brand preference. Paulus and Frank [62] postulated that this region plays a key role in preference judgments, while other authors presented data challenging this hypothesis [63]. McClure et al. [64] examined the brand preference for Pepsi and Coca-Cola drinks by means of fMRI. Finding that in blind tastings, no difference between the response in the brain appeared. However, in open tastings (when subjects could see the brand), limbic structures like the hippocampus and the DLPFC showed enhanced activity, presumably according to brand preference. One conclusion of this experiment is that preference is processed in different brain areas depending on the source of information: the VMPFC is active when preferences were based on sensory information only (taste), while the hippocampus, the DLPFC and the midbrain showed enhanced response when judgments were based on both sensory inputs and the brand. This study, among others, suggests the importance of emotionalizing for the success of a brand [54] and hints at subconscious and purely emotional aspects involved in consumption behavior.

Purchasing is a behavior at least partly determined by the reward system. The pros (reward of buying) and cons (displeasure of paying) have to be weighed up against each other in the sense of a hedonic competition between pleasure and pain [65]. Purchasing acts are preceded by an activation of the nucleus accumbens, which correlates with product preferences, while high prices can lead to an increase of insula activation in the sense of an anticipation of loss [66]. An increase in the BOLD (blood oxygen level dependent) response measured by fMRI in the insula cortex can further precede a negative product choice [66-70]. Thus, preferred brands can be seen as a reward stimulus, and may impair strategic reasoning, probably by a reduced activity of the DLPFC [71]. These preferred brands also seem to activate the reward system more than others [72], while the price of a product directly affects neural reward signals through an increased expectation [73]. Read in conjunction with the previously mentioned article of Knutson et al. [66] a high price can therefore either lead to an anticipation of loss, or to a reward through a high anticipation of utility.

A paradigm commonly used in animal research is conditioned preference. Here, a preference for a neutral stimulus is created by rewards. Johnsrude et al. [74] adapted this approach to human volunteers with unilateral anterior temporal lobe resections, and by doing so created evidence for a role of the amygdala in reward conditioning. A relevant question in this context concerns the degree to which unconscious stimuli can influence behavior [75]. Although there are theories regarding how brand preference is built over time [76], a functional brain imaging study about how brand preference can be conditioned by marketing tools such as advertising has, to the best of our knowledge, not been realized yet. While such a study is certainly challenging concerning the experimental design, it would be an interesting field for future research. In fact, recent fMRI-studies [77,78] indicate celebrity endorser credibility has a modulating effect on product preferences and memory. Celebrity endorsement is a widely used technique in advertising, hence the results of these fMRI-studies could build the basis for a more detailed investigation of advertising’s effects on both product and brand preferences.

To sum up, through the study of purchasing acts and brand preference, general and consumer neuroscience have gained significant knowledge about the reward system, frontal brain regions and their relevance to decision-making. Although there are lesional studies on brand preference and purchasing behavior, a research gap seems to exist, as how neurological diseases affect behavior and decisions in this context.

### Implications for neurology

The reward system is related to a set of behavioral anomalies that are frequently found in neurological diseases, like impulsive-compulsive disorders, including pathological gambling and compulsive buying. As there are both neurologists and consumer neuroscientists involved in research of these behavioral patterns, we think that these contexts offer an opportunity for interdisciplinary research. Pathological gambling is characterized by a loss of control over gambling, deception about the extent of one’s involvement with gambling, family and job disruption, theft, and chasing losses, or the effort to win back money lost while gambling [79]. It is frequent in Parkinson’s disease [80], restless legs syndrome [81], frontotemporal dementia [82], epilepsy [83] and Huntington’s disease [84] and might be the consequence of a neurodegenerative or iatrogenic impairment of reward pathways [85,86]. Pathological gambling is also associated with a reduced activation of the mesolimbic reward system in functional brain imaging [87]. It has already been suggested that psychiatry should adopt findings from neuroeconomics, especially in pathological gambling. ‘…Experimental paradigms derived from NE [neuroeconomics], such as economic exchange games, can be usefully applied to understand psychiatric disorders…’ [88]. We argue that behavioral neurologists should investigate patients suffering from a neurological disease with a higher incidence of pathological gambling using game paradigms of neuroeconomics and paradigms involving brands and purchasing acts of ‘consumer neuroscience’ to learn more about the underlying pathophysiology.

Compulsive buying is a highly debated disorder in the psychiatric field, as its classification as a behavioral addiction or an impulse control disorder is still unclear [89]. Compulsive buying is defined as ‘a tendency to be preoccupied with buying that is revealed by repetitive buying and a lack of impulse control over buying’, with an incidence of 5.8% in the United States [90]. It is considered to be related to the reward system [91]. A higher incidence for this behavior has been reported in patients suffering from Parkinson’s disease [80] and frontotemporal dementia [92]. Further, there is a co-occurrence between depression and impulsive-compulsive buying [93], which supports the theory of an impaired reward system in depressed patients [94]. A recent consumer neuroscience study was able to show a difference in the activation of the reward and loss/pain avoidance system between compulsive and non-compulsive buyers. The former showed a higher activity in the nucleus accumbens and a lower activation of the insula during the presentation of a product and its price than non-compulsive buyers [95]. A study about the responsiveness to brands and advertising of neurological patients suffering from compulsive buying behavior could further advance knowledge in behavioral neurology.

### Trust

Trust is a basic human phenomenon, essential for humans if they are to live among unknown others, and therefore is vital for the functioning of modern societies [96,97]. Multiple definitions of trust exist, but most somehow refer to trust as a behavior [98]. Trust behavior involves the voluntary placement of resources at the disposal of a trustee with no enforceable commitment from the trustee. This situation can either be beneficial for both sides, if the trustee reciprocates, or lead to loss for the trustor if the trustee is opportunistic. Trust thereby involves the risk of betrayal. Recent research indicates that distrust is unlikely to be simply the absence of trust, but a distinct phenomena itself, which makes it possible to have a certain degree of trust and distrust at the same time [99]. However, one of the most challenging tasks in research involving human behavior is the operationalization of trust/distrust. In neuroeconomic research the trust game and the evaluation of trustworthiness of faces are common methods [100,101]^a^.

A detailed review of literature on trust is well beyond the present scope. In what follows then, we give an overview of biologically-orientated literature on trust. For greater detail we refer readers to a number of reviews that can be read in conjunction with the present piece (e.g. [102-104]).

Trusting an unknown person requires an individual to perform a number of stepwise evaluations. Each of these steps is associated with distinct brain areas.

•*Trustworthiness evaluation*: By visual perception of key anatomic features of the other person’s face his or her trustworthiness is assessed. This can lead to uncertainty, ambiguity or fear. During this process the amygdala and the insula cortex show activation in fMRI scans [105-107].

•*Prediction of the other person*’*s future action*: At this stage, questions as to the likelihood of trust reciprocation, deception, prior knowledge of this person, or prior experience of trusting unknowns are evaluated. Here, theory-of-mind regions such as the paracingulate and the medial prefrontal cortex, as well as memory regions (e.g. amygdala and hippocampus) are active [108,109].

•*Calculation of future reward*: Here, the neurobiological reward system is relevant, as the individual assesses the likely reward of their trusting behavior. This system is discussed above (see also e.g. [106,107,110]).

•*Processing of cognitive conflict* is associated with activation in the anterior cingulate cortex [104-106]. In trust situations this area is active, because the risk of betrayal and the possible reward of a beneficial outcome have to be weighed up against each other.

Besides these specific brain areas, several neurotransmitters and hormones modulate trusting behavior (for a review see [104]). Oxytocin, a neuropeptide that plays an important role in social approach behavior, has been found to be associated with trustworthiness [111] and to increase trust when administered intranasally [112]. Oxytocin leads to an increase of dopamine levels [113], and dopamine is thought to be the main neurotransmitter of the reward system [114], which plays an important role in trust (see above). Recent literature suggests, that the prosocial effects of oxytocin might be context dependent in the sense, that oxytocin acts predominantly on behavior towards members participating in a group in contrast to out-group members [115-117]. Cortisol, a stress hormone, has only recently been associated with trust and seems to play an antagonistic role to oxytocin [118]. Further, a gender difference in trust has been proposed, and several surveys show that men trust more than women, (e.g. [119]). Women also exhibit different brain activation patterns in a trustworthiness evaluation task [120], and trust related brain areas (e.g. caudate nucleus) differ in size between the genders [121]. Furthermore, gender dimorphisms for distrust have been reported [122].

In marketing research, consumer trust is a key focus. The perceived trustworthiness of brands is seen as the main basis for brand loyalty, which indicates a certain purchase consistency and brand performance [123,124]. Furthermore, research involving trust in advertisements, as well as trust in online environments (e.g. offers and websites) has flourished in recent years [120,125-127]. Trust in relationships between marketing operatives (e.g. industrial purchasers and sellers) is also a key theme in research [128,129]. However, even though trust is commonly cited as a major research topic in consumer neuroscience (e.g. [29]), experimental data in this area has mostly been acquired by scientists involved in neuroeconomics and decision neuroscience (e.g. [130]).

### Implications for neurologists

In recent years, economically-orientated sciences have shed light on the neurobiology of interpersonal trust through questionnaires and gaming paradigms. Medical research at the same time has focused on the important role that trust plays in the patient-physician relationship, as health outcomes of several diseases have been shown to be associated with trust in physicians [131,132]. Several studies also showed a positive impact of a patient’s trust in physicians on patient satisfaction, therapy adherence and continuity of care [133-135]. Being comforting and caring, demonstrating competency, answering questions, and explaining diagnosis and therapy to the patient has been shown to increase trust [136]. Multiple neurological diseases lead to an impairment of trust-relevant brain areas and/or a disequilibrium of associated hormones. Depression leads, on the one hand, to structural changes in the caudate nucleus and other trust-related brain areas and, on the other hand, to hypercortisolemia [137,138], and should therefore theoretically impair trust on the basis of these two mechanisms. As the reward system is part of the brain’s trust network, it seems plausible that diseases affecting the dopaminergic pathways could also impair trust behavior. Further, there are several neurological diseases affecting frontal brain regions, e.g. frontotemporal dementia and other neurodegenerative diseases as well as certain types of epilepsy, that could lead to lower trust. Empirical behavioral data (e.g. using the trust-game or paradigms, where trust of sale-offers and brands is assessed) is needed to test this hypothesis and can be seen as an opportunity for a fruitful collaboration of neurologists or psychiatrists with researchers in consumer neuroscience. A transfer of knowledge would certainly be profitable for both sides, as it would lead, on the one hand, to new insights into the neurobiology of trust. On the other hand, neurologists could identify diseases leading to lower trust in physicians and deduce guidelines to improve communication with, and therapy adherence of, these patients.

### Ethical aspects from a neurological perspective

Marketing-related topics such as target marketing or consumer vulnerability have traditionally elicited concerns leading to vital scientific and public discussions about the fundamentals of marketing from an ethical perspective (see, e.g., [139]). Ethical evaluations of alternative concepts, models and methodologies applied in marketing have created a discourse in both industry and society. Especially, when it comes to the marketing of pharmaceutical products [140].

As the field of consumer neuroscience and neuromarketing is still new, a comprehensive ethical discussion is vital. In keeping with this, there is a steadily growing number of studies dealing with the ethical aspects of neuromarketing. Potential ethical dilemmas covered in such work include whether technology such as neuroimaging should be employed in an effort to maximize profit [141], and also whether the findings of neuromarketing research can be seen as a violation of individual consumer rights such as privacy [67]. Notwithstanding the common confusion over commercial and scientific approaches to neuromarketing as discussed previously, consumer neuroscience on the other hand has to deal with similar ethical problems as other neuroscientific fields (for an introduction to general neuroethics we recommend [142]).

The question of whether neuroscientific methods should be used for the sole purpose of increasing profit can be seen as the starting point of any ethical consideration on the subject of neuromarketing [141]. However, any attempt to commercialize neuroscience should be of interest to neurologists. From a medical perspective, doctors participating in neuromarketing could lead to a loss of prestige of physicians in general [21] or to the occurrence of conflicts of interest. In particular, publication bias, the phenomenon of positive results being published more frequently than negative results, plays a role in any industry-sponsored research [143]. Reports suggesting that industry may alter, obstruct, or stop publication of negative results have been published [144,145]. These ethical problems that occurred in studies sponsored by the pharmaceutical industry might also manifest in neuromarketing studies.

In an ethical sense, neuromarketing should thoroughly be evaluated based on the potential added-value it might have for product improvement (for example by better knowledge of the consumer's preferences), compared to the sole purpose of maximizing profit [67]. Long-term entrepreneurial success is a primary objective of most economic models. With this in mind, it is natural that it is also the aim of scientific marketing research to discover as much as possible about consumer behavior in order to be able to derive recommendations for improved economic actions. Therefore, it is necessary to instigate a detailed ethical discussion about marketing research and practice and ethical standards [146-150], that includes marketing scientists, practitioners, ethicists and possibly neurologists, who could add methodological knowledge and experience in ethical aspects of clinical research to the discussion. These standards, on which all parties consent, should be applicable to marketing research, applied neuromarketing, and scientific research in the field of consumer neuroscience.

In clinical science, standards of how to protect study participants and provide security of personal data have long been established. For example, ethical guidelines based on the Helsinki Declaration have been institutionalized [151]. However, since they are not classed as medical research, it is possible that some neuromarketing studies do not comply with any ethical declarations. Although the majority of methodologies applied within the frame of neuromarketing are not physically invasive, detailed information provided prior to participation, and written consent given for the use of the results exclusively as stated, are compulsory [67]. All these items should be explicitly stated in a set of rules [152].

The growing media coverage of the topic of neuromarketing has predominantly covered the commonly-feared idea of ‘mind reading of consumers’ private thoughts and the location of the so-called ‘buy-button in the brain’. These misconceptions have their roots primarily in false promises given by commercial agencies. However, as of 2012, such a ‘buy-button’ has not been found, and it seems unlikely that such a thing exists in a scientific sense. In fact, despite consumer fears over neuromarketing, current marketing practice at the point of sale in traditional retail or consumer data transactions within the field of data mining and analytics [153] are likely to be far more manipulative than neuroscientific experiments could ever be, given the complexities of the human brain. Even so, while these commonly-feared ideas seem futuristic, and far beyond the limits of current technology, in light of increasingly fast technological development (and widespread public fears), such a discussion is legitimate [154-163]. An ethical discourse like this would benefit from the expert knowledge and experience of neurologists. It is important in particular for neurologists to enter this debate, and help clarify what type of information current brain-imaging methodology is realistically able to provide and how this information might affect society.

Of special interest to neurologists is the use of neuromarketing practice on children and minorities, as well as ill, disabled, or disadvantaged/powerless individuals. Most authors agree that they need special protection [152] and argue that biological disorders must not be misused by being targeted by specifically-confined marketing activities [67].

The entirety of ethical considerations related to brain imaging in general is also relevant to consumer neuroscience. This includes two major issues, both of which have been subject to ongoing debate among neurologists in general [164-167]; first, how to tackle unexpected pathological findings, that are true for about 1% of the population [168]. Second, issues concerning communicating the findings as completely and truthfully as possible to the public audience [169]. In neuromarketing and consumer neuroscience, functional brain imaging is more common than structural MRI. Even so, as Illes [170] argues: ‘We must ask, for example, whether all studies of normative neurobehavioral phenomena are ethically acceptable. How might social or racial biases affect applications of the technology, the conditions under which imaging is performed, or the way interpretations are made? What does a statistically normal activation pattern of moral behavior really mean, and, by extension, what would the implication of an abnormal brain activation pattern be in a healthy person normally (i.e., within predicted behavioral or physiological norms) performing a task that involves moral judgment, deception, or even sexual responsiveness.’

As a consequence of this ethical debate there are initiatives arising to attempt the creation of standards for the use of neurological methods in marketing. On the one hand, commercial suppliers of neuromarketing methods are, under the pressure of public attention, willing to accept the rules of academic research [171] and on the other hand more and more researchers, like the group of Plassmann et al. [20], publish recommendations for the academic community (e.g. avoid redundant and irrelevant information, employ rigorous experimental setups and establish standards). There are also initiatives establishing rules and guidelines for commercial neuromarketing studies. For instance, in 2011, ESOMAR published ‘36 questions to help commission neuroscience research’ [172]. Another initiative to be mentioned here is the ‘NMSBA Code of ethics for the application of neuroscience in business’ published by NMSBA in 2012 [173]. In addition to these initiatives, we propose the establishment of a registry of companies using neuroimaging in a commercial setting as well as an ethics committee, to take an oversight role regarding the studies run by these companies.

### Implications for neurologists

The ethical implications of neuromarketing and consumer neuroscience are important, because neurologists entering this field must have a basic knowledge in this area, due to the high media presence and possible public critique [174]. We argued earlier for a differentiated view, and proposed the terms ‘neuromarketing’ and ‘consumer neuroscience’, as there are both commercial and scientific applications of neuroscientific methods in a marketing context. With this in mind, it is interesting to note that a recently-published study reports that neurologists were favorable towards neuromarketing, and agreed upon it not being a manipulative way of selling unnecessary goods and services [175]. However, although most methods used in neuromarketing have a low risk profile, there is research emerging using more invasive forms of neurological methodology, such as transcranial magnetic or direct current stimulation [176,177]. As these instruments are frequently used in neurological research, we strongly believe that the entrance of neurologists to the ethical debate around neuromarketing would be beneficial. Disabled persons have already been identified as a group for target marketing [178], and as such it only seems to be a matter of time until neurological patients come into focus in the same way. As we have shown in previous sections, several brain systems that are essentially regulating the reaction to key marketing tools such as brands and advertising are affected by neurological disorders. Therefore, neurological patients might show special consumer vulnerability. To the best of our knowledge there is no empirical data as to how neurological patients react to marketing measures. The results of such studies could be very helpful in initiating an interdisciplinary discussion about a set of standards and codes of conduct for commercial marketing actions on our patients.

## Summary

Consumer neuroscience has gained considerable insights in basic functions of the human brain, through application of neuroscientific methods to marketing research questions. These findings have found a broad audience in the scientific community of economists, biologists and psychologists. There are also neurologists and psychiatrists involved in neuromarketing and consumer neuroscience, although the general medical neuroscientific community has only recently started to draw its attention to the findings of this field of research and how they can contribute to psychiatry [37].

The intention of this paper was to start a similar discussion in the neurological community. We think that especially the field of behavioral neurology could profit from collaboration with economists and marketing researchers, as the neurobiology of behavior is a common interest and there is theoretical evidence that behavioral symptoms of neurological diseases could affect consumer behavior and economic decision-making.

In this article we gave readers an introduction into scientific and commercial applications of neuroscientific methods in marketing. We argued for a differentiated terminology, naming commercial applications ‘neuromarketing’ and scientific applications ‘consumer neuroscience’. Further, we identified a number of key areas where neurologists can gain further insights into the pathophysiology of neurological diseases and correlated behavioral symptoms through an examination of consumption behavior:

First, we think that studies using game paradigms could help to gain further insights into the underlying pathophysiology of pathological gambling in Parkinson’s disease, frontotemporal dementia, epilepsy, and Huntington’s disease.

Second, we identified compulsive buying as a common interest in neurology and consumer neuroscience. Paradigms commonly used in consumer neuroscience could be applied to patients suffering from Parkinson’s disease and frontotemporal dementia to advance knowledge of this important behavioral symptom.

Third, trust research in the medical context lacks empirical behavioral and neuroscientific evidence. Neurologists entering this field of research could profit from the extensive knowledge of the biological foundation of trust that consumer neuroscientists have gained.

Fourth, neurologists could contribute significantly to the ethical debate about invasive methods in neuromarketing and consumer neuroscience. Further, neurologists should investigate biological and behavioral reactions of neurological patients to marketing and advertising measures, as they could show special consumer vulnerability and be subject to target marketing.

## Endnotes

^a^ The trust game involves two players each receiving an amount of money (i.e. 10€). The rules are simple: Player one can freely decide how much of the given amount he wants to send to player 2. Every dollar sent is tripled. Player 2 can then decide how much of the tripled money to keep and how much to send back to Player 1. Classic game theory predicts that player 2 will not send any money back and therefore player 1 will not send any money in the first place. But this is not what was observed empirically. On average players sent 5.16$ and counterparts reciprocated in about one third of the cases by sending back more than they received [100].

## Competing interests

The authors declare that they have no competing interests.

## Authors’ contributions

AJ elaborated the conception and the general structure of the manuscript and drafted it. MK was also involved in the conception, editing and drafting of the article. NL, LC and GR critically revised the draft and wrote parts of the manuscript. All authors have read and approved the final version of the manuscript.

## Pre-publication history

The pre-publication history for this paper can be accessed here:

http://www.biomedcentral.com/1471-2377/13/13/prepub

## References

[B1] Frazzetto G Anker S Neuroculture Nat Rev Neurosci 2009 10 815 821 19841632 10.1038/nrn2736

[B2] Churchland PS Neurophilosophy: toward a unified science of the mind 1986 Cambridge, MA: MIT Press

[B3] Ashbrook J Neurotheology: the working brain and the work of theology Zygon J Sci Theol 1984 19 331 350

[B4] Tallis R Aping mankind: neuromania, darwinitis and the misrepresentation of humanity 2011 London: Acumen

[B5] Senior C Lee N Butler M Perspective: organizational cognitive neuroscience Orga Sci 2011 22 3 804 815 10.1287/orsc.1100.0532

[B6] Camerer CF Loewenstein G Prelec D Neuroeconomics: why economics needs brains Scand J Econ 2004 106 3 555 579 10.1111/j.0347-0520.2004.00377.x

[B7] Glimcher PW Rustichini A Neuroeconomics: the consilience of brain and decision Science 2004 306 447 452 10.1126/science.1102566 15486291

[B8] Zak PJ Neuroeconomics Philos Trans R Soc Lond B Biol Sci 2004 359 1737 1748 15590614 10.1098/rstb.2004.1544 PMC1693452

[B9] Braeutigam S Neuroeconomics-from neural systems to economic behaviour Brain Res Bull 2005 67 355 360 10.1016/j.brainresbull.2005.06.009 16216681

[B10] Fehr E Fischbacher U Kosfeld M Neuroeconomic foundations of trust and social preferences: initial evidence Am Econ Rev 2005 95 346 351 10.1257/000282805774669736 29125275

[B11] Kenning P Plassmann H NeuroEconomics: an overview from an economic perspective Brain Res Bull 2005 67 343 354 10.1016/j.brainresbull.2005.07.006 16216680

[B12] Sanfey AG Loewenstein G McClure SM Cohen JD Neuroeconomics: cross-currents in research on decision-making Trends Cogn Sci 2006 10 108 116 10.1016/j.tics.2006.01.009 16469524

[B13] Smidts A Kijken in het brein: over de mogelijkheden van neuromarketing 2002 Dutch: Inaugural Address Erasmus University: ERIM EIA-12-MKT

[B14] Lee N Chamberlain L Neuroimaging and psychophysiological measurement in organizational research: an agenda for research in organizational cognitive neuroscience Ann NY Acad Sci 2007 1118 18 42 10.1196/annals.1412.003 17717097

[B15] Dawson ME Schell AM Filion DL Cacioppo JT, Tassinary LG, Bernston GG The electrodermal system Handbook of psychophysiology 2000 Boston: Cambridge: University Press 200 223

[B16] Ohme R Reykowska D Wiener D Choromanska A Analysis of neurophysiological reactions to advertising stimuli by means of EEG and galvanic skin response measures J Neurosci Psychol Econ 2009 2 21 31

[B17] Glimcher PW Camerer CF Fehr E Poldrack RA Glimcher PW, Camerer CF, Fehr E, Poldrack RA Introduction: a brief history of neuroeconomics Neuroeconomics: decision making and the brain 2009 Amsterdam: Academic 1 12

[B18] Reimann M Schilke O Weber B Neuhaus C Zaichkowsky J Functional magnetic resonance imaging in consumer research: a review and application Psychol Mark 2011 28 6 608 637 10.1002/mar.20403

[B19] Lee N Senior C Butler MJR The domain of organizational cognitive neuroscience: theoretical and empirical challenges J Manag 2012 38 4 921 931

[B20] Plassmann H Zoega Ramsoy T Milosavljevic M Branding the brain – a critical review and outlook J Consum Psychol 2012 22 18 36 10.1016/j.jcps.2011.11.010

[B21] Fisher CE Chin L Klitzman R Defining neuromarketing: practices and professional challenges Harv Rev Psychiatry 2010 18 4 230 237 10.3109/10673229.2010.496623 20597593 PMC3152487

[B22] Dumit J Is it me or my brain? depression and neuroscientific facts J Med Humanit 2003 24 35 47 10.1023/A:1021353631347

[B23] Hubert M Kenning P A current overview of consumer neuroscience J Consum Behav 2008 7 272 292 10.1002/cb.251

[B24] Singer E They know what you want New Sci 2004 15460578

[B25] Thompson J They don’t just want your money, they want your brain London Independent on Sunday 2005

[B26] Dias D A ‚Buy Button’ in your brain? National Post 2006

[B27] Fullerton RA “A virtual social H-bomb”: the late 1950s controversy over subliminal advertising Journal of Historical Research in Marketing 2010 2 2 166 173 10.1108/17557501011042533

[B28] Pratkanis AR Greenwald AG Recent perspectives on unconscious processing: still no marketing applications Psychol Mark 1988 5 4 339 355

[B29] Lee N Broderick AJ Chamberlain L What is neuromarketing? a discussion and agenda for future research Int J Psychophysiol 2007 63 2 199 204 10.1016/j.ijpsycho.2006.03.007 16769143

[B30] Becker WJ Cropanzano R Sanfey AG Organizational neuroscience: taking organizational theory inside the neural black box J Manag 2011 37 933 961

[B31] Herman S Selling to the brain Glob Cosmet Ind 2005 173 5 64 66

[B32] Huang G The economics of brains Technol Rev 1998 108 5 74 76

[B33] Lovel J Nader group slams Emory for brain research 2003 Chronicle: Atlanta Business

[B34] Editorial. Brain scam? Nat Neurosci 2004 7 10 1015 10.1038/nn1004-1015 15452565

[B35] Laybourne P Lewi D Neuromarketing: the future of consumer research? Admap 2005 461 28 30

[B36] Willingham DT Dunn EW What neuroimaging and brain localization can do, cannot do, and should not do for social psychology J Personal Soc Psychol 2003 85 4 662 671 10.1037/0022-3514.85.4.662 14561120

[B37] Sharp C Monterosso J Montague PR Neuroeconomics: a bridge for translational research Biol Psychiatry 2012 72 2 87 92 10.1016/j.biopsych.2012.02.029 22727459 PMC4096816

[B38] Shiv B Fedorikhin A Heart and mind in conflict: the interplay of affect and cognition in consumer decision making J Consum Res 1999 26 278 292 10.1086/209563

[B39] Shiv B Emotions, decisions, and the brain J Consum Psychol 2007 17 3 174 178 10.1016/S1057-7408(07)70025-6

[B40] Cohen JD The vulcanization of the human brain: a neural perspective on interactions between cognition and emotion J Econ Perspect 2005 19 4 3 24 10.1257/089533005775196750

[B41] American academy of neurologyhttp://www.aan.com/globals/axon/assets/2726.pdf

[B42] Lee N Senior C Butler MJR Leadership research and cognitive neuroscience: the state of this union Leadersh Q 2011 23 213 218

[B43] Peterson RL The neuroscience of investing: fMRI of the reward system Brain Res Bull 2005 67 391 7 10.1016/j.brainresbull.2005.06.015 16216685

[B44] Spencer H Principles of psychology 1880 New York: Appleton Press

[B45] Bechara A Damasio AR The somatic marker hypothesis: a neural theory of economic decision Games and Economic Behavior 2005 52 336 372 10.1016/j.geb.2004.06.010

[B46] Buchel C Dolan RJ Classical fear conditioning in functional neuroimaging Curr Opin Neurobiol 2000 10 219 233 10.1016/S0959-4388(00)00078-7 10753800

[B47] O’Doherty J Kringelbach ML Rolls ET Hornak J Andrews C Abstract reward and punishment representations in the human orbitofrontal cortex Nat Neurosci 2001 4 95 102 10.1038/82959 11135651

[B48] Ploghaus A Tracey I Gati JS Clare S Menon RS Matthews PM Rawlins JNP Dissociating pain from its anticipation in the human brain Science 1999 284 1979 1981 10.1126/science.284.5422.1979 10373114

[B49] Rolls ET The brain and emotion 1999 Oxford: Oxford University Press

[B50] Rolls ET The orbitofrontal cortex and reward Cereb Cortex 2000 10 284 294 10.1093/cercor/10.3.284 10731223

[B51] Schultz W Tremblay L Hollerman JR Reward processing in primate orbitofrontal cortex and basal ganglia Cereb Cortex 2000 10 272 283 10.1093/cercor/10.3.272 10731222

[B52] Schultz W Dayan P Montague RA Neural substrate of prediction and reward Science 1997 275 1593 1599 10.1126/science.275.5306.1593 9054347

[B53] Erk S Martin S Walter H Emotional context during encoding of neutral items modulates brain activation not only during encoding but also during recognition NeuroImage 2005 26 3 829 838 10.1016/j.neuroimage.2005.02.045 15955493

[B54] Breiter HC Aharon I Kahneman D Dale A Shizgal P Functional imaging of neural responses to expectancy and experience of monetary gains and losses Neuron 2001 30 619 639 10.1016/S0896-6273(01)00303-8 11395019

[B55] Gray JA Stahl SM, Iversen SD, Goodman ED The neuropsychology of emotion and personality Cognitive neurochemistry 1987 Oxford: Oxford University Press 171 90

[B56] Fournier S Consumers and their brands: developing relationship theory in consumer research J Consum Res 1998 24 343 53 10.1086/209515

[B57] Priester JR Nayakankuppam D Fleming MA Godek J The A2sc2 model: the influence of attitudes and attitude strength on consideration and choice J Consum Res 2004 30 574 84 10.1086/380290

[B58] Park CW MacInnis DJ Priester J Eisingerich AB Iacobucci D Brand attachment and brand attitude strength: conceptual and empirical differentiation of two critical brand equity drivers J Mark 2010 74 1 17

[B59] Miller EK Cohen JD An integrative theory of prefrontal cortex function Annu Rev Neurosci 2001 24 167 202 10.1146/annurev.neuro.24.1.167 11283309

[B60] Ballard IC Murty VP Carter RM MacInnes JJ Huettel SA Adcock RA Dorsolateral prefrontal cortex drives mesolimbic dopaminergic regions to initiate motivated behavior J Neurosci 2011 31 10340 10346 10.1523/JNEUROSCI.0895-11.2011 21753011 PMC3182466

[B61] Fellows LK Farah MJ The role of ventromedial prefrontal cortex in decision making: judgment under uncertainty or judgment per se? Cereb Cortex 2007 17 2669 2674 10.1093/cercor/bhl176 17259643

[B62] Paulus MP Frank LR Ventromedial prefrontal cortex activation is critical for preference judgments NeuroReport 2003 14 1311 1315 12876463 10.1097/01.wnr.0000078543.07662.02

[B63] Santos JP Seixas D Brandão S Moutinho L Investigating the role of the ventromedial prefrontal cortex in the assessment of brands Front Neurosci 2011 5 77 21687799 10.3389/fnins.2011.00077 PMC3108388

[B64] McClure SM Li J Tomlin D Cypert KS Montague LM Montague PR Neural correlates of behavioral preference for culturally familiar drinks Neuron 2004 44 379 87 10.1016/j.neuron.2004.09.019 15473974

[B65] Prelec D Loewenstein GF The red and the black: mental accounting of savings and debt Mark Sci 1998 17 4 28 10.1287/mksc.17.1.4

[B66] Knutson B Rick S Wimmer GE Prelec D Loewenstein G Neural predictors of purchases Neuron 2007 53 147 156 10.1016/j.neuron.2006.11.010 17196537 PMC1876732

[B67] Ariely D Berns GS Neuromarketing: the hope and hype of neuroimaging in business Nat Rev Neurosci 2010 11 284 292 10.1038/nrn2795 20197790 PMC2875927

[B68] Erk S Spitzer M Wunderlich AP Galley L Walter H Cultural objects modulate reward circuitry NeuroReport 2002 13 2499 2503 10.1097/00001756-200212200-00024 12499856

[B69] Peterson RL Insight the investor’s brain: the power of mind over money 2007 Hoboken: Wiley

[B70] Plassmann H Kenning P Ahlert D Why companies should make their customers happy: the neural correlates of customer loyalty Adv Consum Res 2007 34 735 739

[B71] Deppe M Schwindt W Kugel H Plassmann H Kennig P Nonlinear responses within the medial prefrontal cortex reveal when specific implicit information influences economic decision making J Neuroimaging 2005 15 171 182 15746230 10.1177/1051228405275074

[B72] Schaefer M Rotte M Favorite brands as cultural objects modulate reward circuit NeuroReport 2007 18 141 145 10.1097/WNR.0b013e328010ac84 17301679

[B73] Plassmann H O’Doherty J Shiv B Rangel A Marketing actions can modulate neural representations of experienced pleasantness Proc Natl Acad Sci USA 2008 105 1050 1054 10.1073/pnas.0706929105 18195362 PMC2242704

[B74] Johnsrude IS Owen AM White NM Xhao WV Bohbot V Impaired preference conditioning after anterior temporal lobe resection in humans J Neurosci 2000 20 2649 2656 10729345 10.1523/JNEUROSCI.20-07-02649.2000 PMC6772234

[B75] Kouider S Dehaene S Levels of processing during non-conscious perception: a critical review of visual masking Philos Trans R Soc Lond B Biol Sci 2007 362 1481 857 875 10.1098/rstb.2007.2093 17403642 PMC2430002

[B76] Rangel A Camerer C Montague PR A framework for studying the neurobiology of value-based decision making Nat Rev Neurosci 2008 9 7 545 556 10.1038/nrn2357 18545266 PMC4332708

[B77] Klucharev V Smidts A Fernández G Brain mechanisms of persuasion: how ‘expert power’ modulates memory and attitudes SCAN 2008 3 353 366 19015077 10.1093/scan/nsn022 PMC2607059

[B78] Stallen M Smidts A Rijpkema M Smit G Klucharev V Fernández G Celebrities and shoes on the female brain: the neural correlates of product evaluation in the context of fame J Econ Psychol 2010 31 802 811 10.1016/j.joep.2010.03.006

[B79] American Psychiatric Association Diagnostic and statistical manual of mental disorders (4th ed.) 1994 Washington, DC: American Psychiatric Association

[B80] Voon V Hassan K Zurowski M de Souza M Thomsen T Fox S Lang AE Miyasaki J Prevalence of repetitive and reward-seeking behaviors in Parkinson’s disease Neurology 2006 67 1254 1257 10.1212/01.wnl.0000238503.20816.13 16957130

[B81] Tippmann-Peikert M Park JG Boeve BF Shepard JW Silber MH Pathologic gambling in patients with restless legs syndrome treated with dopaminergic agonists Neurology 2007 68 301 303 10.1212/01.wnl.0000252368.25106.b6 17242339

[B82] Nakaaki S Murata Y Sato J Shinagawa Y Hongo J Tatsumi H Mimura M Furukawa TA Impairment of decision-making cognition in a case of frontotemporal lobar degeneration (FTLD) presenting with pathologic gambling and hoarding as the initial symptoms Cogn Behav Neurol 2007 20 121 125 10.1097/WNN.0b013e31804c6ff7 17558256

[B83] Cavanna AE Mula M Strigaro G Servo S Tota G Barbagli D Collimedaglia L Viana M Cantello R Monaco F Clinical correlates of pathological gambling symptoms in patients with epilepsy Epilepsia 2008 49 1460 1464 10.1111/j.1528-1167.2008.01586.x 18479402

[B84] De Marchi N Morris M Mennella R La Pia S Nestadt G Association of obsessive-compulsive disorder and pathological gambling with Huntington’s disease in an Italian pedigree: possible association with Huntington’s disease mutation Acta Psychiatr Scand 1998 97 62 65 10.1111/j.1600-0447.1998.tb09964.x 9504705

[B85] Bergh C Eklund T Sodersten P Nordin C Altered dopamine function in pathological gambling Psychol Med 1997 27 473 475 10.1017/S0033291796003789 9089839

[B86] Grosset KA Macphee G Pal G Stewart D Watt A Davie J Grosset DG Problematic gambling on dopamine agonists: not such a rarity Mov Disord 2006 21 2206 2208 10.1002/mds.21110 17013907

[B87] Reuter J Raedler T Rose M Hand I Gläscher J Buechel C Pathological gambling is linked to reduced activation of the mesolimbic reward system Nat Neurosci 2005 8 147 148 10.1038/nn1378 15643429

[B88] Ross D Sharp C Vuchinich R Spurrett D Midbrain mutiny: the picoeconomics and neuroeconomics of disordered gambling 2008 Cambridge, MA: The MIT Press

[B89] Hollander E Allen A Is compulsive buying a real disorder, and is it really compulsive? (editorial) Am J Psychiatry 2006 163 1670 1672 10.1176/appi.ajp.163.10.1670 17012670

[B90] Ridgway NM Kukar-Kinney M Monroe KB An expanded conceptualization and a new measure of compulsive buying J Consum Res 2008 35 622 639 10.1086/591108

[B91] Grant JE Brewer JA Potenza MN The neurobiology of substance and behavioural addictions CNS Spectr 2006 11 924 30 17146406 10.1017/s109285290001511x

[B92] McKhann GM Albert MS Grossman M Miller B Dickson D Trojanowski JQ Clinical and pathological diagnosis of frontotemporal dementia: report of the work group on frontotemporal dementia and Pick’s disease Arch Neurol 2001 58 1803 1809 10.1001/archneur.58.11.1803 11708987

[B93] Lejoyeux M Hourtané M Adès J Compulsive buying and depression J Clin Psychiatry 1995 56 1 38 7836343

[B94] Nestler EJ Carlezon WA Jr The mesolimbic dopamine reward circuit in depression Biol Psychiatry 2006 59 1151 1159 10.1016/j.biopsych.2005.09.018 16566899

[B95] Raab G Elger CE Neuner M Weber B A neurological study of compulsive buying behaviour J Consum Policy 2011 34 401 413 10.1007/s10603-011-9168-3

[B96] Pedersen CA How love evolved from sex and gave birth to intelligence and human nature J Bioecon 2004 6 39 63

[B97] Zak PJ Knack S Trust and growth Econ J 2001 111 470 295 321 10.1111/1468-0297.00609

[B98] Coleman JS Foundations of social theory 1990 Cambridge, London: Harvard University Press

[B99] Lewicki RJ McAllister DJ Bies RJ Trust and distrust: new relationships and realities Acad Manag Rev 1998 23 438 458

[B100] Berg J Dickhaut J McCabe K Trust, reciprocity, and social-history Games and Economic Behavior 1995 10 122 142 10.1006/game.1995.1027

[B101] McCabe K Smith V A two person trust game played by naive and sophisticated subjects Proc Natl Acad Sci USA 2002 97 3777 3781 10725349 10.1073/pnas.040577397 PMC16316

[B102] Zak PJ Kurzban R Matzner WT The neurobiology of trust Annals of the NY Academy of Sciences 2004 1032 224 227 10.1196/annals.1314.025 15677415

[B103] Fehr E On the economics and biology of trust J Eur Econ Assoc 2009 7 235 266 10.1162/JEEA.2009.7.2-3.235

[B104] Riedl R Javor A The biology of trust: integrating evidence from genetics, endocrinology, and functional brain imaging Journal of Neuroscience, Psychology, and Economics 2012 5 63 91

[B105] Winston JS Strange BA O’Doherty J Dolan RJ Automatic and intentional brain responses during evaluation of trustworthiness of faces Nat Neurosci 2002 5 3 277 283 10.1038/nn816 11850635

[B106] Baumgartner T Heinrichs M Vonlanthen A Fischbacher U Fehr E Oxytocin shapes the neural circuitry of trust and trust adaptation in humans Neuron 2008 58 639 650 10.1016/j.neuron.2008.04.009 18498743

[B107] King-Casas B Tomlin D Anen C Camerer CF Quartz SR Montague PR Getting to know you: reputation and trust in a two-person economic exchange Science 2005 308 5718 78 83 10.1126/science.1108062 15802598

[B108] Krueger F McCabe K Moll J Kriegeskorte N Zahn R Strenziok M Heinecke A Grafman J Neural correlates of trust Proc Natl Acad Sci USA 2007 104 50 20084 20089 10.1073/pnas.0710103104 18056800 PMC2148426

[B109] Frith U Mind blindness and the brain in autism Neuron 2001 32 969 979 10.1016/S0896-6273(01)00552-9 11754830

[B110] Delgado MR Frank RH Phelps EA Perceptions of moral character modulate the neural systems of reward during the trust game Nat Neurosci 2005 8 11 1611 1618 10.1038/nn1575 16222226

[B111] Zak PJ Kurzban R Matzner WT Oxytocin is associated with human trustworthiness Horm Behav 2005 48 5 522 527 10.1016/j.yhbeh.2005.07.009 16109416

[B112] Kosfeld M Heinrichs M Zak PJ Fischbacher U Fehr E Oxytocin increases trust in humans Nature 2005 435 7042 673 676 10.1038/nature03701 15931222

[B113] Shahrokh DK Zhang TY Diorio J Gratton A Meaney MJ Oxytocin-dopamine interactions mediate variations in maternal behavior in the rat Endocrinology 2010 151 5 2276 86 10.1210/en.2009-1271 20228171 PMC2869254

[B114] Ikemoto S Panksepp J The role of nucleus accumbens dopamine in motivated behavior: a unifying interpretation with special reference to reward-seeking Brain Res Rev 1999 31 6 41 10.1016/S0165-0173(99)00023-5 10611493

[B115] Stallen M De Dreu CKW Shalvi S Smidts A Sanfey AG The herding hormone: Oxytocin stimulates in-group conformity Psychol Sci 2012 23 11 1288 1292 10.1177/0956797612446026 22991128

[B116] DeDreu CKW Greer LL Handgraaf MJJ Shalvi S Van Kleef GA Baas M The neuropeptide oxytocin regulates parochial altruism in intergroup conflict among humans Science 2010 328 1408 1411 10.1126/science.1189047 20538951

[B117] De Dreu CKW Shalvi S Greer LL Van Kleef GA Handgraaf MJ Oxytocin motivates non-cooperation in intergroup conflict to protect vulnerable in-group members PLoS One 2012 7 11 e46751 10.1371/journal.pone.0046751 23144787 PMC3492361

[B118] Takahashi T Ikeda K Ishikawa M Kitamura N Tsukasaki T Nakama D Kameda T Interpersonal trust and social stress-induced cortisol elevation NeuroReport 2005 16 197 199 10.1097/00001756-200502080-00027 15671877

[B119] Alesina A La Ferrara E Who trusts others? Journal of Public Economic 2002 85 207 234 10.1016/S0047-2727(01)00084-6

[B120] Riedl R Hubert M Kenning P Are there neural gender differences in online trust? an fMRI study on the perceived trustworthiness of ebay offers MIS Q 2010 34 397 428

[B121] Cosgrove KP Mazure CM Staley JK Evolving knowledge of sex differences in brain structure, function, and chemistry Biol Psychiatry 2007 62 8 847 855 10.1016/j.biopsych.2007.03.001 17544382 PMC2711771

[B122] Zak PJ Borya K Matzner WT Kurzban R The neuroeconomics of distrust: sex differences in behavior and physiology Am Econ Rev 2005 95 360 363 10.1257/000282805774669709 29125276

[B123] Lau G Lee S Consumers’ trust in a brand and the link to brand loyalty J Mark-Focus Manag 1999 4 341 70

[B124] Chaudhuri A Holbrook MB The chain of effects from brand trust and brand affect to brand performance: the role of brand loyalty J Mark 2001 65 81 93 10.1509/jmkg.65.2.81.18255

[B125] Pavlou PA Consumer acceptance of electronic commerce: integrating trust and risk with the technology acceptance model Int J Electron Commer 2003 7 101 134

[B126] Gefen D Reflections on the dimensions of trust and trustworthiness among online consumers Database 2002 33 38 53

[B127] Dimoka A What does the brain tell us about trust and distrust? evidence from a functional neuroimaging study MIS Q 2010 34 373 396

[B128] Morgan RM Hunt SD The commitment-trust theory of relationship marketing J Mark 1994 58 20 38

[B129] Das K Relationship marketing research (1994–2006): an academic literature review and classification Marketing Intelligence and Planning 2009 27 3 326 363 10.1108/02634500910955236

[B130] Rilling JK Sanfey AG The neuroscience of social decision-making Annu Rev Psychol 2011 62 23 48 10.1146/annurev.psych.121208.131647 20822437

[B131] Lee YY Lin JL The effects of trust in physician on self-efficacy, adherence and diabetes outcomes Soc Sci Med 2009 68 1060 1068 10.1016/j.socscimed.2008.12.033 19162386

[B132] Nguyen GC Laveist TA Harris ML Datta LW Bayless TM Brant SR Patient trust-in-physician and race are predictors of adherence to medical management in inflammatory bowel disease Inflamm Bowel Dis 2009 15 1233 1239 10.1002/ibd.20883 19177509 PMC2799328

[B133] Thom DH Campbell B Patient-physician trust: an exploratory study J Fam Pract 1997 44 169 76 9040520

[B134] Thom DH Ribisl KM Stewart AL Luke DA Validation of a measure of patients’ trust in their physician: the trust in physician scale Med Care 1999 37 510 17 10.1097/00005650-199905000-00010 10335753

[B135] Safran DG Taira DA Rogers WH Kosinski M Ware JE Tarlov AR Linking primary care performance to outcomes of care J Fam Pract 1998 47 213 20 9752374

[B136] Thom DH Stanford Trust Study Physicians Physician behaviors that predict patient trust J Fam Pract 2001 50 323 328 11300984

[B137] Gibbons J McHugh P Plasma cortisol in depressive illness J Psychiatry Res 1962 1 162 171 10.1016/0022-3956(62)90006-7 13947658

[B138] Sheline YI 3D MRI studies of neuroanatomic changes in unipolar major depression: the role of stress and medical comorbidity Biol Psychiatry 2000 48 791 800 10.1016/S0006-3223(00)00994-X 11063975

[B139] Smith NC Cooper-Martin E Ethics and target marketing: the role of product harm and consumer vulnerability J Mark 1997 61 3 1 20 10.2307/1251786

[B140] Sillup GP Porth SL Ethical issues in the pharmaceutical industry: an analysis of US newspapers International Journal of Pharmaceutical and Healthcare Marketing 2008 2 3 163 180 10.1108/17506120810903953

[B141] Madan CR Neuromarketing: the next step in market research? Eureka 2010 1 1

[B142] Farah MJ Neuroethics: the practical and the philosophical Trends Cogn Sci 2005 9 34 40 10.1016/j.tics.2004.12.001 15639439

[B143] Bekelman JE Li Y Gross CP Scope and impact of financial conflicts of interest in biomedical research JAMA 2003 289 454 65 10.1001/jama.289.4.454 12533125

[B144] Rennie D Thyroid storm JAMA 1997 277 1238 1243 10.1001/jama.1997.03540390068038 9103350

[B145] Bodenheimer T Uneasy alliance: clinical investigators and the pharmaceutical industry N Engl J Med 2000 342 1539 1544 10.1056/NEJM200005183422024 10816196

[B146] Murphy PE Ethics in advertising: review, analysis, and suggestions Journal of Public Policy & Marketing 1998 17 2 316 31920169798

[B147] Laczniak GR Marketing ethics: onward toward greater expectations Journal of Public Policy & Marketing 1993 12 1 91 9620169798

[B148] Robin DP Reidenbach RE Searching for a place to stand: toward a workable ethical philosophy for marketing Journal of Public Policy & Marketing 1993 12 1 97 10520169798

[B149] Gaski JF Does marketing ethics really have anything to say? – a critical inventory of literature Journal of Business Ethics 1999 18 315 334

[B150] Brinkmann J Business and marketing ethics as professional ethics. Concepts, approaches and typologies Journal of Business Ethics 2002 41 159 177 10.1023/A:1021318710382

[B151] World Medical Association Helsinki declarationhttp://www.wma.net/en/30publications/10policies/b3/index.html

[B152] Murphy ER Illes J Reiner PB Neuroethics of neuromarketing J Consum Behav 2008 7 293 302 10.1002/cb.252

[B153] New York timeshttp://www.nytimes.com/2012/06/17/technology/acxiom-the-quiet-giant-of-consumer-database-marketing.html?pagewanted=all

[B154] Kulynych J Legal and ethical issues in neuroimaging research: human subjects protection, medical privacy, and the public communication of research results Brain Cogn 2002 50 345 357 10.1016/S0278-2626(02)00518-3 12480482

[B155] Tovino SA Functional neuroimaging and the law: trends and directions for future scholarship Am J Bioeth 2007 7 44 56 17849344 10.1080/15265160701518714

[B156] Tovino SA The confidentiality and privacy implications of functional magnetic resonance imaging J Law Med Ethics 2005 33 844 850 10.1111/j.1748-720X.2005.tb00550.x 16686253

[B157] Illes J Empirical neuroethics. Can brain imaging visualize human thought? Why is neuroethics interested in such a possibility? EMBO Rep 2007 8 Spec No 57 60 10.1038/sj.embor.7401007 PMC3327527 17726446

[B158] Illes J Racine E Imaging or imagining? a neuroethics challenge informed by genetics Am J Bioeth 2005 5 5 18 16036688 10.1080/15265160590923358 PMC1506750

[B159] Greely HT Illes J The social effects of advances in neuroscience: legal problems, legal perspectives Neuroethics: defining the issues in theory, practice, and policy 2006 Oxford: Oxford University Press 245 264

[B160] Alpert S Brain privacy: how can we protect it? Am J Bioeth 2007 7 70 73 17849353 10.1080/15265160701518862

[B161] Appelbaum PS Law & psychiatry: the new lie detectors: neuroscience, deception, and the courts Psychiatr Serv 2007 58 460 462 10.1176/appi.ps.58.4.460 17412845

[B162] Grimes A Are we listening and learning? understanding the nature of hemispherical lateralisation and its application to marketing Int J Mark Res 2006 48 439 458

[B163] Lindstrom M Buyology: truth and lies about what we buy 2010 New York, NY: Broadway Business

[B164] Heinemann T Hoppe C Listl S Spickhoff A Elger CE Incidental findings in neuroimaging: ethical problems and solutions Dtsch Arztebl 2007 104 27 A1982 7

[B165] Heinemann T Hoppe C Weber B Elger CE Ethically appropriate handling of incidental findings in human neuroimaging research Clin Neuroradiol 2009 19 3 242 243 10.1007/s00062-009-1111-5 19705081

[B166] Hentschel F Von Kummer R Concentrating on the next version: reply to the letter by Thomas Heinemann et al. To the guest editorial of frank hentschel and rüdiger von kummer Clin Neuroradiol 2009 19 3 244 10.1007/s00062-009-1112-4 19705081

[B167] Hentschel F VonKummer R Response of the german society of neuroradiology to the guideline “ethically appropriate reaction to incidental imaging findings in brain research”, suggested by thomas heinemann, institut für wissenschaft und ethik, and christian hoppe, klinik für epileptologie, universität bonn, germany, on january 9, 2009 Clin Neuroradiol 2009 19 2 108 110 10.1007/s00062-009-1003-8 19636500

[B168] Illes J Kirschen MP Edwards E Stanford LR Bandettini P Cho MK Ford PJ Glover GH Kulynych J Macklin R Michael DB Wolf SM Incidental findings in brain imaging research Science 2006 311 783 784 10.1126/science.1124665 16469905 PMC1524853

[B169] Racine E Bar-Ilan O Illes J FMRI in the public eye Nat Rev Neurosci 2005 6 2 159 164 10.1038/nrn1609 15685221 PMC1524852

[B170] Illes J Neuroethics in a new era of neuroimaging Am J Neuroradiol 2003 24 1739 1741 14561594 PMC7976301

[B171] Tarran B Eight firms to take part in ARF neuroscience validation studyhttp://www.research-live.com/news/technology/eight-firms-to-take-part-in-arf-%20neuroscience-validation-study/4003667.article

[B172] ESOMARhttp://www.esomar.org/news-and-multimedia.php?pages=&idnews=57

[B173] NMSBAhttp://www.neuromarketing-association.com/ethics

[B174] Ruskin G Commercial alert asks feds to investigate neuromarketing research at Emory universityhttp://www.commercialalert.org/news/news-releases/2003/12/commercial-alert-asks-feds-to-investigate-neuromarketing-research-at-emory-university

[B175] Eser Z Bahar Isin F Tolon M Perceptions of marketing academics, neurologists, and marketing professionals about neuromarketing J Mark Manag 2011 27 854 868 10.1080/02672571003719070

[B176] Fecteau S Knoch D Fregni F Sultani N Boggio P Pascual-Leone A Diminishing risk-taking behavior by modulating activity in the prefrontal cortex: a direct current stimulation study J Neurosci 2007 27 12500 5 10.1523/JNEUROSCI.3283-07.2007 18003828 PMC6673338

[B177] Camus M Halelamien N Plassmann H Shimojo S O’Doherty J Camerer C Rangel A Repetitive transcranial magnetic stimulation over the right dorsolateral prefrontal cortex decreases valuations during food choices Eur J Neurosci 2009 30 1980 1988 10.1111/j.1460-9568.2009.06991.x 19912330

[B178] Friedman HH Lopez-Pumarejo T Friedman LW Frontiers in multicultural marketing: the disabilities market Journal of International Marketing and Marketing Research 2007 32 1 25 39

